# Other Schemes and Views

**Published:** 1920-03-13

**Authors:** 


### CONTRIBUTIONS FROM PATIENTS.

### OTHER SCHEMES AND VIEWS.

The following letters reveal the alternatives proposed at various hospitals and the views of those responsible for their management as to the practicability or otherwise of the London Hospital proposal (*a*) generally and (*b*) as applied to the individual institution:—

### Mr. A. Whitham, Chairman, Beckett Hospital, Barnsley.

At the end of 1919 our accumulated debt was over £17,000, owing to deficiencies of income during war time. Three weeks ago our Mayor (Colonel Raley, J.P.) made an appeal on our behalf for £20,000 to clear off the debt and place us on a better financial basis. £13,000 has already been given, and we hope to have the balance paid up by end of next April. We are appealing also for £4,500 a year increased subscriptions to keep us going. There are over forty collieries in our area, and the miners have agreed to pay 1s. per week, stopped from their wages, to help us. This will increase our income £3,000 a year. The other £1,500 required is being got from increased subscriptions from workmen in other industries and new annual subscribers. We have no doubt about realising our wishes, in which case we shall be financially sound. Under these circumstances I could not advocate asking for 10s. a week as a maintenance fee for in-patients.

### Rev. R. H. M. Bouth, Hon. Treasurer, Cheltenham General Hospital.

The plan advocated by myself, and which we are now trying to carry out, is to induce all the wage-earners of the town to allow 2d. a week to be deducted from their wages for the benefit of the hospital. This is not, of course, a new plan, but hitherto it has not prevailed in this district, and should it prove successful our financial situation would be greatly eased. If this scheme cannot be adopted, I see nothing for it but to make a charge covering cost of food, at any rate. The former plan I much prefer.

### Mr. Frederick Acton, Chairman, General Hospital, Nottingham.

I have grave doubt of the practicability or desirability of the suggested request of 10s. per week from each in-patient towards his, or her, maintenance. Assuming all the in-patients at this hospital were to comply (which is most improbable), the financial aid resulting would be very small as compared with our present expenditure, whilst the effect upon our large and increasing voluntary support would be disastrous as regards income, and would tend to destroy that spirit of human sympathy and zealous aid which are now so gratifying and beneficial. The moral effect of any interference with the present purely

**Contributions from Patients—(continued).**

voluntary system might, in my judgment, be the start of its entire collapse. I am still hopeful that the result of the efforts now being made in this district, by both employers and employed, will be such increased income as will enable us to continue our good work without diminution of beds and without our having to adopt lines other than those which have ever been the basis of our institution.

An alternative suggestion to that of the proposed 10s. per week demand from patients is that each voluntary hospital now needing the extra financial help adopt organised and vigorous methods, such as we are pursuing here, of bringing home to all classes their duty in their own interest of doing their best to provide what is needed and the consequence of their failure to do so. Should that fail and some other course be necessary, I suggest it might be in the direction of an "annexe" to the hospital for paying patients, such annexe being managed by the existing staff and committees, but made entirely self-supporting and having separate accounts and being in no way a tax upon the main part of the hospital, which should be continued and supported on the present strictly voluntary lines, and any profit made upon such paying annexe should go to aid that larger part which would continue, as now, voluntary and free.

I write as chairman of this large voluntary hospital, and as one who, in such capacity or otherwise, has had a close and active experience for over twenty-six years, and also as one who for several years has been closely associated, as their president, with a large Hospital Saturday Organisation composed of the employed.

**Mr. E. D. H. Buckley, Chairman, Salisbury Infirmary.**

The Committee of this hospital have been debating the subject of payment (for maintenance only) for some weeks, and we have now called for a special court of our Governors to enable us to charge 10s. 6d. per patient per week in future. Of course, really necessitous cases will be free as hitherto, but it has been felt for some time, both in the hospital and outside, that a large majority of our cases can pay and are really glad to help support the infirmary.

**Mr. Nigel Hanbury, Treasurer, Paddington Green Children's Hospital.**

My own personal opinion is that there are many difficulties in carrying out the proposal put forward by the London Hospital, as far as children's hospitals and similar institutions are concerned. At the same time, some drastic changes will have to be made in the near future if our hospitals are to be maintained efficiently.

**Mr. S. S. Cole, Secretary, Royal Devon and Exeter Hospital.**

Having noted the decision of the London Hospital to invite their patients to contribute towards their maintenance, I beg to state that such a scheme might work very well in London, where the patients are distributed and collected from a vast area, but it is very doubtful whether it would be a success in the provinces, particularly in cities like Exeter, where there are very few factories, and where the hospital is not supported by the working class to such an extent as in larger towns. I would, however, mention that ever since the introduction of the National Insurance Act it has been the custom at this hospital, when insured patients are admitted, to interview them with the idea of obtaining their sanction to a proportion of their sickness benefit moneys being transferred to the funds as a slight return for their board, and, by this means, the hospital has benefited on an average to the extent of £250 a year, such sum being, in the majority of cases, most willingly given. It will be interesting to watch the effect of the proposition which the London Hospital is now about to undertake, and if the principle is generally approved by the provincial hospitals, I have no doubt that this institution will be prepared to follow suit.

**Mr. Walter Francombe, Secretary, Walsall General Hospital.**

I do not think that the 10s. a week contribution scheme would be a success in Walsall, but I do think a greater effort should be made generally to encourage hospital patients to contribute more liberally than at present. The percentage of donations from grateful patients is far too small, especially now that their means have so substantially increased. By improved organisation between almoner and canvasser better results may be obtained. But as a definite scheme for increasing funds, I am strongly in favour of a vigorous campaign for obtaining largely increased contributions from the working classes. The *idée fixé* that a 1d. per week is the correct contribution should be dispelled for ever. At a time when the average cost of an in-patient is nearly £10, it takes the contributor fifty years to pay his share. If there were no voluntary hospitals nearly every working man would be willing to pay £1 a year for the certainty of being properly treated in case of illness, and I think, therefore, that the working classes should be educated to look on 6d. per week as a suitable amount to give to the hospital. In Walsall a Workpeople's Organisation Committee exists, and a direct appeal has been made to employers to obtain the sanction of their workpeople for the deduction of 3d. a week from their wages, the total amount to be forwarded by cheque at the end of the financial year. There is a firm in this town which adds 50 per cent. to whatever its employees contribute, and if all employers viewed their responsibilities in the same fashion we should have no fear for the future of Walsall General Hospital.

**Mr. Godfrey Hamilton, Secretary, National Hospital for the Paralysed and Epileptic.**

This hospital has for some time adopted the principle of payments by patients, according to means, towards the cost of their maintenance. It has been felt by us that it was unfair that the hospital should relieve the family of the entire cost of food, etc., and thus in practice make them a substantial gift, apart from the question of treatment of the patient, which is the proper function of the hospital. These payments are quite easily collected, and the system works with great smoothness. We have here also two contributing wards, in which the patients make a uniform payment of, at present, two guineas per week, which fee, however, it is probable will be increased in the near future.

**Captain I. H. Johnson, Secretary, Royal Westminster Ophthalmic Hospital.**

In relation to the "paying principle" being adopted by the London hospitals, I have to inform you that the whole matter comes up for discussion at my April Board meeting. As to whether any change will be made I do not, of course, know; if such a scheme is adopted, it will mean altering our charter. The individual opinion of the members of the Board is against it at present, as this special hospital, although finding it difficult to obtain funds for maintenance purposes, is not in dire financial straits. Undoubtedly the general hospitals are passing

**Contributions from Patients**—(*continued*).

through a bad time; the special hospitals, from what I gather from other officers, are not doing so badly.

The whole question of patients' payments opens up a wide field for discussion. For instance, the charges made at a general hospital (*i.e.*, the London, for example, at 10s. per week) would not be anything like sufficient at a special hospital, and I may mention that at the Royal Westminster Ophthalmic Hospital each in-patient costs £5 10s. 1d., based on the statistical tables for the year 1919, or £2 8s. 4d. per week.

My contention is that a part voluntary and part payment scheme will not answer; run our institutions on a commercial basis, with staffs adequately paid, and the probabilities are that the problem of financial support will be solved. Moreover, there is another factor which does not seem to be considered—viz., the question of the medical and surgical staff. There is no doubt that in the near future some remuneration will have to be given for their services. I also consider that considerable economies could be effected in expenditure by the extensive use of co-operative buying. In conclusion, the necessitous poor would be always treated free of charge, the remainder pay, and thus do away with the old-fashioned and obsolete term "charity."

**Mr. D. D. Kirkaldy Willis, Secretary, West End Hospital.**

I have for some time caused in-patients, on their admission to our free wards, to be asked to pay 5s. or 10s. per week, although the payment is not insisted upon. It must be borne in mind that, coming into hospital, patients are relieved of the cost of food in their private homes. Except in certain cases, when their illness deprives them of all income, they should be able to contribute.

**The Secretary of a Children's Hospital.**

In reply to yours of March 4, I think that all patients attending hospital should contribute according to their means. Something will have to be done to increase the incomes of the voluntary hospitals to enable them to carry on, but the amount likely to be collected from patients will probably not be sufficient to meet the deficiency. I advocate the system of paying wards for those who can pay the full or part cost of their treatment, and free wards for those who are unable to pay anything. The members of the staff attending paying patients should receive a fee for their services. Failing this meeting the difficulty, the hospitals would, in my opinion, have to be subsidised by the State, or out of the rates, or else taken over altogether.

